# The therapeutic significance of mutational signatures from DNA repair deficiency in cancer

**DOI:** 10.1038/s41467-018-05228-y

**Published:** 2018-08-17

**Authors:** Jennifer Ma, Jeremy Setton, Nancy Y. Lee, Nadeem Riaz, Simon N. Powell

**Affiliations:** Department of Radiation Oncology, Memorial Sloan Kettering Cancer Center, New York, NY 10065 USA

## Abstract

Cancer is fundamentally a disease of the genome and inherited deficiencies in DNA repair pathways are well established to increase lifetime cancer risk. Computational analysis of pan-cancer data has identified signatures of mutational processes thought to be responsible for the pattern of mutations in any given cancer. These analyses identified altered DNA repair pathways in a much broader spectrum of cancers than previously appreciated with significant therapeutic implications. The development of DNA repair deficiency biomarkers is critical to the implementation of therapeutic targeting of repair-deficient tumors, using either DNA damaging agents or immunotherapy for the personalization of cancer therapy.

## Abstract (web summary)

Targeting DNA repair-deficient tumors is one of the most promising therapeutic strategies in cancer research; however, accurately predicting which tumors will respond can be a challenge. Here the authors present a review of the current state of knowledge in DNA repair deficiency across human cancers.

## Introduction

Genomic instability is an enabling hallmark of cancer and facilitates the acquisition of genetic events that ultimately promote oncogenic transformation^1^. This instability can occur at the level of chromosomal arm changes, large- and small-scale copy number changes, specific patterns of mutations, or some combination of all three types of events. Initial evidence of the importance of genomic stability in oncogenesis originated from hereditary syndromes with marked increase in cancer risk such as Lynch Syndrome and hereditary breast and ovarian cancer (HBOC) syndrome, which were ultimately linked to germline mutations in key DNA repair genes^2,3^. These hereditary syndromes are thought to only account for 3–5% of colon cancer and 5–7% of breast cancer respectively^2,4^.

**Fig. 1:**
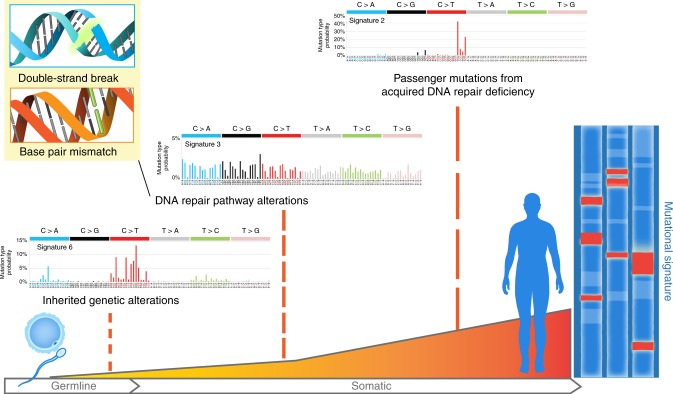
An individual’s unique mutational signature is a record of the types of DNA alterations sustained throughout their lifetime and can be studied to identify unique patterns of etiology-specific alterations, including carcinogens or DNA repair pathway defects, the latter of which can be inherited or acquired during oncogenesis. Mutational signatures adapted from COSMIC with permission (http://cancer.sanger.ac.uk)^118^

In the past decade, large-scale sequencing and genomic characterization efforts have helped better characterize the frequency of genomic instability and DNA repair deficiencies in cancer. Unlike defects in other pathways, the phenotypic consequences of a defect in a DNA repair pathway are detectable from sequencing data (Fig. 1). Careful analysis of the patterns of mutations and copy number changes in a tumor has allowed for the delineation of a number of mutational processes responsible for genomic instability in individual tumors^5^. These analyses have suggested that defects in pathways responsible for genomic instability may occur at a significantly higher frequency than previously appreciated. Further recent epidemiologic evidence has suggested that up to two-thirds of the mutations in cancer are thought to be caused by errors during DNA replication^6^.

Here we will review the main DNA repair pathways altered in cancer and new methods used to detect specific repair pathway defects, specifically focusing on sequencing-based methods. We will review how these data have informed the prevalence of repair defects across cancer and helped identify the various methods by which repair pathways are inactivated. Identification of a specific DNA repair pathway defect could facilitate a precision oncology approach permitting selection of therapies that can take advantage of particular DNA repair defects utilizing a synthetic lethal approach. Lastly we will review how various DNA repair defects influence the micro-environmental phenotype of a tumor, which may in turn influence a tumor’s vulnerability to micro-environmentally directed therapies, such as immunotherapy.

## Pathways for repair of specific DNA lesions

The machinery to maintain genomic integrity has been divided into pathways that are responsible for repairing specific lesions that occur in DNA, although significant cross talk occurs between these pathways^7^. These include pathways responsible for repairing double-strand breaks, for repairing base damage or adducts by base excision repair (BER) or bulky adducts by the nuclear excision repair (NER) pathway, correction of base mismatches via mismatch repair (MMR), or direct repair of direct damage to bases by methyl-guanine methyl-transferase (MGMT; see Box 1 for additional details). Each of these pathways has been reviewed in depth elsewhere^7,8^. Double-stranded breaks (DSBs) are a potent tumorigenic type of DNA lesion. The main pathways involved in DSB repair are homologous recombination (HR) and non-homologous end joining (NHEJ) and each pathway has an alternative pathway, namely, single-strand annealing (SSA) and alternative end joining respectively. Among these DSB repair pathways, HR is the cell’s highest fidelity method of repairing double-stranded DNA breaks as it uses intact homologous duplex sequence, usually the sister chromatid, as a template and is active only during the S and G2 phases of the cell cycle. The specific lesions generated will influence methods for detecting defects in the pathway and play an important role in the micro-environmental phenotype of the resulting malignancy.

In addition to the known DNA repair pathways, emerging evidence strongly suggests that APOBEC plays an important role in tumorigenesis^9^. APOBEC enzymes are involved in somatic hypermutation and virus protection, and are a common cause of mutation in cancers^10^. APOBEC consists of a family of seven enzymatic DNA cytosine deaminases responsible for somatic hypermutation, class-switching recombination, and RNA viral defense. Although their precise roles are still unclear, they catalyze the hydrolytic conversion of cytosine to uracil in single-stranded DNA, which results in C>T transition. In turn, the uracil is removed by BER and, when the site is abasic, synthesis adds a cytosine opposite, resulting in a C>G transversion. Thus, this combination of mutagenic repair accounts for the APOBEC mutational signature.

### Box 1 Defects in key DNA repair pathways generate unique mutational signatures detectable in individual tumors, including base excision repair, single-strand break repair, direct repair with MGMT, nucleotide excision repair, and double-strand break repair (DSBs). The mechanism of the three pathway choices in the repair of DSBs (HR, NHEJ, and SSA) are depicted

NHEJ non-homologous end joining, HR homologous recombination, SSA single-strand annealing

The three main pathways involved in DSB repair are homologous recombination (HR), non-homologous end joining (NHEJ), and single-strand annealing (SSA). HR involves strand invasion by one strand of the sister chromatid, D-loop formation, and DNA synthesis, resulting in either crossover or non-crossover products depending upon the method of resolution of the Holliday junction. A crossover product is the result of the exchange of genetic material between homologous strands resulting in recombinant DSB products. SSA is a DSB break repair pathway that anneals homologous repeats to bridge DSB ends. However, the SSA pathway is mutagenic as the DNA sequence between the two repeats is lost, resulting in deletions^121^. Break-induced replication (BIR) is a mechanism of repair for DSBs found at replication forks due to unwinding by DNA helicase. The BIR pathway also plays a role in telomere length maintenance in the absence of telomerase, preventing telomere shortening in budding yeast^122^.

Mismatch repair (MMR) is a post-replicative repair mechanism, which repairs errors in DNA replication during S phase. Replicative DNA polymerases can occasionally produce base mismatches or create extra-helical nucleotides due to strand slippage during replication^95^. Persistence of these errors due to defective MMR beyond S phase can result in missense or frameshift mutations and microsatellite instability.

Nucleotide excision repair (NER) is a more involved method of single-stranded DNA repair, with over 30 involved proteins and a multi-step process of DNA damage sensing, recruitment of a repair complex, and re-synthesis and ligation of the repaired DNA. It is the main mechanism of repair of UV-induced pyrimidine dimers. It has significant therapeutic implications as the main mechanism of repair of platinum-based agents such as cisplatin, carboplatin, and oxaliplatin. There are several small molecule inhibitors in development for a number of the proteins involved in this pathway, although none have currently demonstrated efficacy in a clinical trial setting.

Direct repair via O6-methylguanine DNA methyltransferase (MGMT*)* is the fastest, simplest, and most common form of single-stranded DNA repair, comprising only one step. MGMT transfers a methyl group from the damaged DNA to itself which results in irreversible MGMT inactivation and degradation. As such, continuous production of MGMT is required to perform direct repair.

## Phenotypic evidence of DNA repair deficiency directly from human cancers

Functional evaluation of aberrations in DNA repair processes in tumors can be determined via in vitro/in vivo testing of tumor material or genomic analysis. Defects in DNA repair processes can lead to specific patterns of mutations or structural alterations and can indicate a defect in a specific repair pathway or can lead to abnormal gene expression patterns.

### Mutational signatures

During the course of their evolution, individual cancers acquire a larger number of somatic mutations and copy number alterations (Fig. 1). Although passenger mutations in cancer genomes were initially thought to be random, it has become increasingly clear that underlying defects in the DNA repair machinery lead to biases in the types of passenger mutations and structural events that are created in tumors. Detection of these biases in sequencing data can help identify underlying repair process that may be defective in a particular tumor.

Before the advent of high-throughput sequencing, the discovery of Lynch Syndrome was a clear indication that the presence of passenger genetic events could indicate a DNA repair deficiency. Lynch syndrome, a cancer predisposition syndrome associated with an elevated risk of colon and endometrial cancers, is now known to be caused by germline mutations in the MMR pathway. MMR plays an important role in correcting insertion/deletion loops that occur during replication and are less likely to be caught by proof-reading domains of DNA replication polymerases, especially at microsatellites^11^. Detection of unstable microsatellites has been used to identify MMR-deficient tumors in colon and endometrial cancers for nearly two decades^12^. Recent pan-cancer extension of this type of analysis has identified low levels of MMR deficiencies in many other malignancies, some not previously thought to be associated with Lynch Syndrome^13^.

The advent of high-throughput genomic assays and the profiling of tens of thousands of tumors has led to the development of signatures (or specific types of DNA lesions; Table 1) that can identify specific DNA repair pathway defects in a similar manner to which microsatellite instability indicates MMR deficiency. Genomic technologies such as array-based comparative genomic hybridization or single-nucleotide polymorphism arrays have been used to identify copy number changes that may associate with an underlying pathway defect. For example, array-based approaches have been used to define a genomic signature of HR deficiency and identified significant changes in copy number profiles between *BRCA1/*2-mutated tumors and those that were wild type in breast cancer. Three different groups identified patterns of allelic imbalance and copy number changes such as sub-telomeric allelic imbalances, large-scale state transitions (LST), and loss of heterozygosity that were associated with HR deficiency^14–16^. Current analysis suggests that these measures of genomic instability have a high negative predictive value (when low, patients are unlikely to respond); however, their positive predictive value remains modest.

**Table 1:**
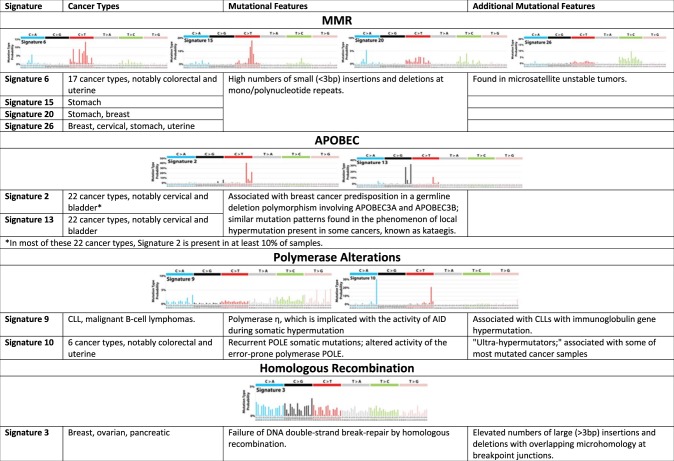
Distinct DNA damage-related mutational signatures by DNA repair pathway^118^

Portions adapted from COSMIC with permission (cancer.sanger.ac.uk)

Signatures within the same pathways commonly co-occur with all other signatures within that pathway

In addition to looking at structural changes, the availability of thousands of tumor genomes or exomes from The Cancer Genome Atlas (TCGA) and others have allowed for the characterization of mutational signatures in tumors. By examining the 6 types of single-nucleotide mutations in addition to the context in which the mutation occurs (i.e., the base preceding the mutated position and the base right after), one can define 96 possible mutation types. This type of analysis leads to a matrix where the rows consist of tumor and the columns the number of mutations belonging to each the aforementioned 96 types of mutations. The underlying processes responsible for generating mutations in these tumors can be deduced using one of several mathematical frameworks, with the most commonly used referred to as non-negative matrix factorization (NMF)^17^. Alexandrov et al.^5^ applied this approach to 7042 tumors and identified 21 different mutational signatures. Some of these signatures were associated with aging or exposure to a specific carcinogen, while some were associated with deficiencies in DNA repair pathways. Now multiple DNA repair pathways, including HR, NER, MMR, and APOBEC, are associated with their own mutational signature, suggesting that the defect in that pathway played a role in oncogenesis in that particular cancer. As previously mentioned, pan-cancer analysis has suggested pathways such as MMR and HR are defective at a low incidence in atypical malignancies, i.e., those not typically associated with their respective hereditary syndromes^13,18^. Still, the tissue specificity for predisposition risk remains perplexing from a purely DNA repair-based perspective, and perhaps suggests that oncogenic risk is influenced by emerging micro-environmental differences from defects in these pathways. The latter may in turn be influenced by tissue subtype (see below).

HR has also been well studied in the context of mutational signatures. In the initial description of mutational signatures, the HR-related signature was termed signature 3, and was associated with a relatively uniform incidence of each of the 96 mutation subtypes and an increase in the number of indels^5^. This was presumed to occur due to HR deficiency because its presence was associated with mutations in *BRCA1/2*. Although HR-defective tumors are enriched in breast, ovarian, and prostate cancer, they also occur in 5-10% of all cancers. In the context of HR-defective cancers, the association with indels especially with micro-homology at the breakpoint makes mechanistic sense as this presumably occurred by error-prone NHEJ and alt-EJ; however, the uniform distribution of mutations remains less clear mechanistically^19^. Translesion synthesis (TLS) polymerases are involved in the repair of endogenous DNA damage at apurinic sites; they preferentially insert adenine resulting in a unique mutational signature with increased C to T alterations, which is not consistent with signature 3^20^. Therefore, daughter-strand gap repair by TLS polymerases, although increased in HR-deficient tumors, is not responsible for the mutational signature observed.

A significant limitation of exome sequencing-based analysis is the limited number of mutations for signature-based analysis and the poor ability to capture structural events for analysis. Nik-Zainal et al.^21^ recently examined whole-genome sequencing of 560 human breast cancers which allowed for more careful examination of structural alterations. They classified structural alterations into 32 subclasses, first, based on whether structural changes occurred in a cluster, second, based on whether they involved a deletion, inversion, or a tandem duplication, and, lastly, based on the size of the structural alteration. Application of a decomposition framework to the matrix of 32 subclasses × 560 cases identified 6 rearrangement signatures. Interestingly, this framework allowed separating out DNA lesions generated from *BRCA1* vs. *BRCA2* deficiency, with the former enriched in rearrangement signature 3 (small tandem duplications). Both *BRCA1/2* deficiencies were linked to rearrangement signature 5, which was associated with an elevated number of deletions less than 1 Mb in size. Although most of the lesions identified still do not have a clear mechanistic association, it was recently demonstrated in vitro that the specificity of tandem duplications in *BRCA1*-mutant carriers arises from a replication-restart bypass mechanism terminated by either end joining or template switching by micro-homology-mediated end joining^22^.

Analyses of structural abnormalities have also revealed a diverse array of other abnormalities in genome maintenance programs, although not all of these are indicative of an ongoing DNA repair defect. For example, chromothripsis is a single cataclysmic event which can result in hundreds of locally clustered chromosomal rearrangements and DNA loss in a limited number of chromosomes^23^. Recent work has suggested this type of event occurs through aberrant isolation of a chromosome in a micronucleus and is not indicative of a traditional ongoing DNA repair defect^24^. Chromoplexy, by contrast, involves complex genomic rearrangements in a smaller number of breakpoints, but engages a number of different chromosomes, resulting in a significant level of rearrangements presumably from a single initiating event. Certain structural rearrangements can be linked to retro-transposon activity in the tumor cell while other structural abnormalities have been associated with ongoing replication defects. For example, fold-back inversions have recently identified a subset of ovarian cancers with a poor prognosis and are thought to be indicative of breakage-fusion-bridge cycles^25^. A recent pan-cancer analysis of whole genomes identified nine different rearrangement signatures, including multiple with possible replication-based etiologies^26^. Whether these newly identified replication-associated defects can be therapeutically targeted in a manner similar to other DNA repair-based defects remains to be determined. Chromosomal instability is also found in human cancers but is generally linked to anaphase defects of chromosomal separation rather than an underlying DNA repair defect.

Although not involved in a classic DNA repair pathway, the APOBEC family of cytidine deaminases have emerged as a significant source of mutagenesis in cancer^27^. Experimental evidence has suggested that deregulation of the APBOEC family of enzymes leads to mutations in (TC(A|T) → (T|G)) sequence context (i.e., deamination of a cytosine occurs in a TCA or TCT context and leads to a subsequent mutation to T or G)^27^. Alexandrov found that signatures associated with presumed APOBEC deregulation (signatures 2 and 13) occurred in nearly 15% of cancers^5^. A focused analysis for enrichment in APOBEC-related mutagenesis in 14 tumor types revealed it to be a prominent source of mutagenesis in bladder, breast, cervical, head and neck cancers, and within breast cancer more prominently in HER2-positive tumors^27^.

Multiple other DNA damage repair pathways have now been linked to a specific mutational signature associated with deficiency. In bladder cancer, where *ERCC2* is recurrently mutated, a mutational signature that displayed a broad pattern of base changes and was similar to signature 5 was associated with mutations in *ERCC2*^28^. Similarly, mutations in polymerases have been associated with their own mutational signatures, including POLE and POLN^5,27^. POLE in particular is strongly associated with C→A mutations in a TCT context or C→T mutations in a TCG context. In addition to instability at microsatellites, MMR is associated with four different mutational signatures, although whether these are more sensitive and/or specific for MMR deficiency remains to be determined^5^. Lastly, studies have found distinct signatures for secondary cancers after ionizing radiation (IR) therapy, suggesting a signature specifically related to repair of double-stranded breaks, with an increased number of small deletions (<100 bp) with micro-homology at the breakpoint^29,30^.

Beyond identifying these patterns of mutational change, it is important to recognize that a mutational or structural signature-based approach to understanding DNA repair in tumors has limitations. Signatures in a tumor represent an archeological history of the tumor during its development and may not reflect whether a pathway is currently deficient in a tumor (i.e., a subsequent genetic event has occurred to restore a particular pathways effect, e.g., a reversion mutation)^31^. The importance of this issue for treatment remains to be determined, but likely will be increasingly important for patients who have received several previous therapies. Identifying subclonal mutations that are produced by the defective pathway may provide a sequencing-based method to circumvent this issue.

### Functional assays

Immunohistochemistry (IHC) or immunofluorescence can be used to visualize nuclear recruitment of DNA damage response-related proteins as a real-time assessment of DNA repair capacity, such as Rad51^32^. IHC allows for the selective imaging of proteins through protein labeling with specific primary antibodies and a fluorescently labeled secondary antibody. These results can then be quantified, providing a readout of the overall repair capacity of the cell. However, they often require assessment after a cytotoxic insult rather than assessment of basal levels.

RAD51 plays a key role in DNA repair via HR as it localizes to sites of DNA damage and forms a nucleoprotein filament on single-stranded DNA, which is readily detectable by standard immunofluorescence. Visualization of Rad51 foci following DNA damage serves as an indicator of HR repair capacity. Low Rad51 foci have been identified in ex vivo studies of breast and ovarian cancer cells, and are predictive of pathological complete response to anthracycline-based chemotherapy^33^. Our group has demonstrated that mutational signatures associated with HR deficiency are associated with Rad51 deficiency, but mutational signatures based on exomes do not appear to capture all cases of HR deficiency^34^.

### Gene expression signatures

Gene expression assays have been reviewed extensively elsewhere and are beyond the scope of this review^35^. They have the benefit of providing real-time expression levels of DNA repair genes, therefore providing a snapshot of current HR status. However, their exact relationship to the repair defect is often obscure and reproducibility has been questioned for these approaches.

## Mechanisms of DNA repair deficiency in cancer

The signature studies above have markedly increased the presumed prevalence of DNA repair defects in cancer. The mechanism by which specific pathways become defective in an individual cancer, however, remains an area of active investigation. Multiple different methods of inactivation have been proposed, including genetic and epigenetic mechanisms.

### Genetic inactivation

The best-characterized mechanism of DNA repair deficiency in cancer is genetic inactivation through germline and/or somatic alteration at the DNA sequence level. Germline inactivation of genes involved in DNA repair is known to cause familial syndromes with cancer predisposition phenotypes, including hereditary nonpolyposis colorectal cancer (HNPCC, also known as Lynch syndrome) and HBOC. In both HNPCC and HBOC—which are caused by defective MMR and HR, respectively—the underlying genetic defect consists of a heterozygous germline allele inherited in autosomal dominant fashion, with transformation thought to require somatic loss of the second wild-type allele. A number of less common hereditary cancer syndromes, including Fanconi anemia, ataxia-telangiectasia, Bloom syndrome, MUTYH-associated polyposis, and xeroderma pigmentosum, are inherited as autosomal recessive disorders and require biallelic germline inactivation for the full cancer predisposition phenotype^36,37^. In addition to these high-penetrance familial syndromes, recent efforts to genotype sporadic tumors on a population scale have shed considerable light on germline mutations associated with more moderate cancer susceptibility and revealed a higher than anticipated prevalence of pathogenic alleles with known roles in DNA repair^38,39^. In a recent pan-cancer analysis, 12% of patients with metastatic cancer were noted to harbor pathogenic germline mutations in known tumor suppressor genes, of which 75% were related to DNA repair^39^. These analyses have also revealed that several variant alleles, previously recognized to cause familial syndromes when inherited as biallelic (homozygous or compound heterozygous) mutations, can result in moderate cancer susceptibility risk when inherited as a single deleterious allele^40–43^. Examples include BRIP1, PALB2, and ataxia-telangiectasia mutated (ATM) variant alleles that produce a Fanconi anemia (BRIP1 and PALB2) or ATM phenotype when inherited in biallelic fashion^41,43^, but result in only moderate cancer susceptibility as a single deleterious allele.

Population-scale sequencing studies have also demonstrated that DNA repair genes undergo somatic inactivation at a significant frequency, not only as a ‘second hit’ among patients with a heterozygous germline mutation, but as biallelic events. Interestingly, a subset of DNA repair-related genes appear to be preferentially affected by somatic alterations (CDK12, BAP1), whereas others are preferentially affected by germline alterations (BRCA1/2, CHEK2, PALB2)^18^. Regardless of whether they occur as germline or somatic events, the majority of altered DNA repair genes appear to require biallelic inactivation for a repair-deficient phenotype to become evident^16,44,45^. Consistent with this notion, biallelic alterations in HR genes, but not monoallelic alterations, have been shown to be associated with HR-related genomic signatures, including LST and signature 3^18,45^. Notable exceptions to this rule do exist however, as evidenced by haploinsufficient or dominant-negative effects of heterozygous mutations in POLD1, POLE, and ERCC2^46,47^.

### Epigenetic inactivation

Epigenetic mechanisms that are known to play a role in the regulation of DNA repair include DNA methylation, histone modification, nucleosome remodeling, and RNA-mediated targeting. Many of these biological processes have been shown to be dysregulated in cancer, leading to transcriptional silencing of DNA repair genes or changes in chromatin dynamics required for DNA repair. Our understanding of such epigenetic processes has substantially increased over the last several years, coincident with the development of technologies to globally study the epigenome and its downstream effects on gene expression and chromatin structure.

### DNA methylation

Genome-wide studies of CpG methylation have demonstrated that between 5 and 10% of normally unmethylated CpG promoter islands are abnormally methylated in human cancer^48^, typically resulting in transcriptional silencing of the associated gene^49^. Somatic loss of function via promoter methylation has been detected for genes involved in the repair of mutagenic and cytotoxic adducts (MGMT)^50^, MMR (MLH1), and HR (BRCA1, RAD51C)^51^.

Perhaps the most direct epigenetic predisposition to genomic instability is the association of the microsatellite instability phenotype with epigenetic silencing of MLH1^52^. MLH1 hypermethylation has been shown to occur as an early event in multi-step tumorigenesis, prior to the appearance of microsatellite instability, as it has been observed in apparently normal colonic epithelium adjacent to colorectal cancers with microsatellite instability (MSI)^53^ and premalignant endometrial lesions preceding the development of an apparent MSI phenotype^54^.

The importance of epigenetic silencing in cancer etiology is similarly illustrated by the example of MGMT promoter methylation, which can lead to development of G→A transition mutations resulting from unrepaired *O*^6^-methylguanine adducts. Similar to MLH1, MGMT promoter methylation appears to occur as an early event in tumorigenesis and has been observed in premalignant polyps^55^. In addition to its role in tumorigenesis, however, MGMT plays a critical role in the cellular response to alkylating agents; its epigenetic silencing via methylation is a strong predictive biomarker for response to alkylating agents in glioblastoma, colorectal cancer, and Hodgkin lymphoma^56–58^.

Epigenetic silencing of BRCA1 and RAD51C via promoter hypermethylation is well characterized in breast and ovarian cancer^59^. BRCA1 (and RAD51C) methylation has been shown to correlate with signature 3^51^ as well as tandem duplication signatures indicative of HR deficiency^21^. Although methylated tumors have HR-defective signature, in both breast and ovarian cancer these particular events do not appear to be associated with response to HR-directed therapy, perhaps suggesting resistance can emerge faster in the setting of epigenetic inactivation. Further, not all methylation leads to significant effects, for example, promoter methylation of BRCA2 and other HR genes does not appear to correlate with expression levels^60^, suggesting gene-specific vulnerabilities to epigenetic silencing that are analogous to those observed with MMR deficiency and MLH1 methylation.

### Other mechanisms

Histone post-translational modifications, including phosphorylation, acetylation, methylation, ubiquitylation, and SUMOylation, are known to play key roles in the DNA damage response including facilitating chromatin remodeling^61,62^. Recently, impaired chromatin remodeling secondary to an oncometabolite has been linked to homologous recombination deficiency. Lu and colleagues^63^ demonstrated that epigenetic changes induced by local accumulation of tricarboxylic acid (TCA) cycle intermediates can lead to impaired repair of IR-induced DSBs. This work suggests that epigenetic changes induced by accumulation of TCA cycle intermediates may represent an exploitable DNA repair deficiency in isocitrate dehydrogenase-mutant cancers^64^.

Several malignancies are virally induced including a subset of head and neck cancer (human papillomavirus (HPV) and Epstein–Barr virus (EBV)), gastric cancers (EBV), and cervical cancers (HPV). Not surprisingly, to facilitate their own replication, viruses are known to manipulate the DNA damage response (DDR) network at multiple steps^65^, including DNA damage sensing by the MRN (Mre11-Rad50-Nbs1) complex^66^ and DDR kinase signaling by ATM and ATR (ATM- and Rad3-related)^67,68^. In addition to inhibition of cell cycle checkpoint function, some viruses have evolved to manipulate DNA damage signaling as a means of replicating their genomes while bypassing origin licensing requirements^69,70^, which has been shown to generate DSBs that are often aberrantly repaired, leading to copy number changes and chromosomal translocations^71^. Further, in some instances viral deregulation of cell cycle checkpoint proteins may have direct effects on DSB repair; for example, E7 interaction with RB1 may have downstream effects on NHEJ via modulation of its role on Ku activity^72^.

## Targeting DNA repair defects with a synthetically lethal approach

**Fig. 2:**
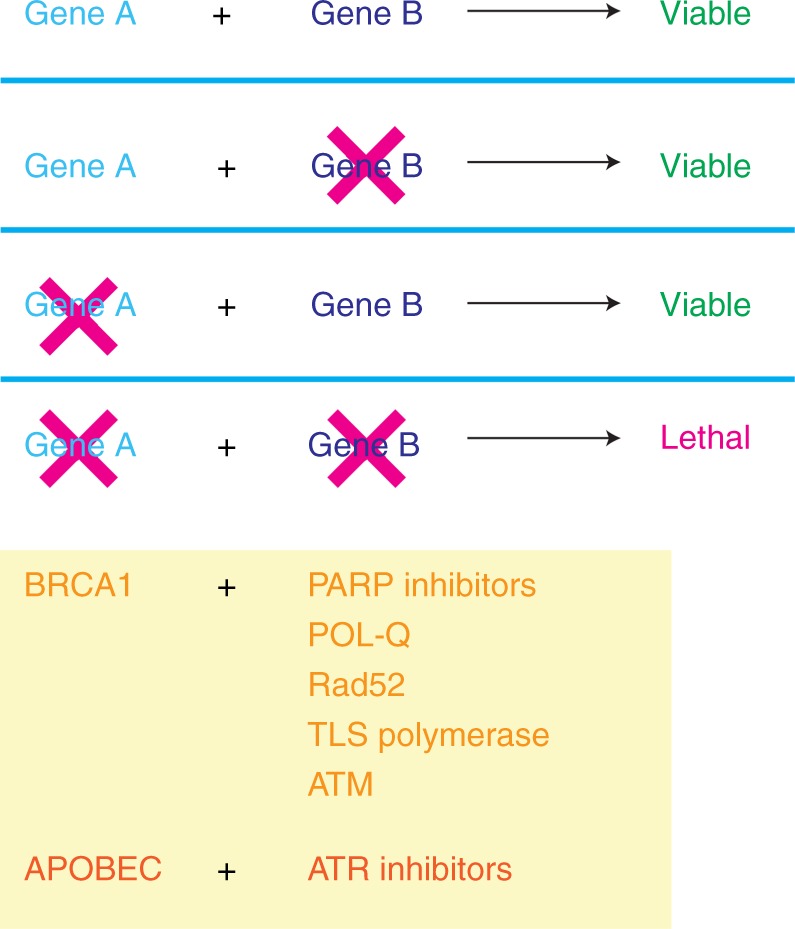
Targeting DNA repair deficiency: mechanism of synthetic lethality of PARP inhibitors and alternative synthetic lethal relationships

Synthetic lethality is the process of cell death resulting from alterations in two or more genes, while alteration of either gene alone is insufficient for cell death (Fig. 2). This mechanism can be exploited therapeutically to target DNA repair-deficient tumors while sparing normal tissue which is DNA repair proficient, as demonstrated by the poly (ADP ribose) polymerase (PARP) inhibitors^73^. Discovery of additional synthetic lethal relationships may provide additional therapeutic targets. In contrast, synthetic viability may mediate PARP inhibitor resistance, whereby a second deficiency may mitigate the impact of the existing DNA repair defects.

*BRCA1* and *BRCA2* play critical roles in HR and mutations in either gene can result in HR deficiency (HRD). PARP1 is a protein required for repairing single-stranded breaks (SSBs); inhibition of PARP1 results in SSBs, which persist and are converted to DSBs during DNA replication. Synthetic lethality between BRCA1 and PARP1 can be exploited through treatment of HR-deficient tumor cells with PARP inhibitors, while sparing normal cells which are HR competent. This approach has so far demonstrated the most significant efficacy in breast and ovarian cancers with known *BRCA1/2* mutations, with advanced FDA (Food and Drug Administration) approval granted for the treatment of BRCA-mutant ovarian and prostate cancers. The prevalence of HRD in cancer has become apparent as PARP inhibitors continue to be tested in additional cancer types.

### Discovery of additional synthetic lethal relationships

The success of PARP inhibitors highlights the therapeutic potential of targeting DNA repair alterations in cancers with HRD. In addition to PARP1, there are three other key proteins synthetically lethal with BRCA1/BRCA2: POLQ, RAD52, and TLS polymerases. POLQ is required for alternative end joining, a back-up pathway in DNA repair-deficient cancers^74^. Rad52 is required for HR in the absence of Rad51, and provides Rad51 function during S phase in Rad52-mediated HR^75^. Lastly, TLS polymerases are involved in post-replication gap repair arising from blocking lesions at replication forks^76^. The lack of TLS polymerases results in the accumulation of daughter-strand gaps that are converted to DSBs in the absence of BRCA1/2. The existence of multiple pathways of synthetic lethality with BRCA defects suggests distinct DNA repair intermediates are formed in cells with HRD. In addition, the genetic context (additional alterations in the cell or tumor) may also control the dependency on the use of any one particular back-up pathway.

Large-scale screens for synthetic lethal interactions have been performed to identify additional therapeutic targets^77,78^. Further investigation of additional cancer types has identified synthetic lethality between PARP and androgen receptor signaling in prostate cancer^79^, and the p53 and phosphatidylinositide 3-kinase/Akt pathways in glioblastomas^80^. Better understanding of the relationships between various pathways will allow for combination therapy with drugs that directly target these back-up pathways.

The APOBEC family of enzymes is a common cause of mutation in cancers and is thought to be a key mutagenic agent in tumor development. This is supported by the presence of APOBEC overexpression in a wide variety of cancers including breast, bladder, lung, colorectal, and ovarian, as compared to its low levels in normal human tissues. Furthermore, preclinical studies suggest that APOBEC3A/B induces replication stress which renders cells sensitive to ATR inhibitors but not to other DNA damaging agents such as ATM inhibitors, suggesting a unique mechanism of replication stress. In fact, ATR appears to halt APOBEC-driven replication stress; in this context ATR inhibitors may allow for continuous APOBEC-related generation of AP sites at replication forks, leading to replication catastrophe^81^.

### Analysis of exceptional responders to traditional cytotoxic therapies to uncover synthetically lethal relationships

DNA damaging agents are a mainstay of cancer treatment, including alkylating agents, IR, and certain targeted biologics. DNA damaging agents have been demonstrated to be more efficacious in a DNA repair-deficient setting in a variety of cancers. Analysis of exceptional responders to these agents may help identify previously opaque repair defects in cancers.

Using synthetic lethal approaches to targeting DNA repair pathways extends beyond HR. A genomic analysis of urothelial carcinomas by the TCGA demonstrated a large number of DNA alterations. *ERCC2*, a gene involved in NER, was identified to be statistically significantly mutated in bladder cancer^82^. The NER pathway detects and repairs bulky adducts and is therefore critical in response to cross-linking agents such as cisplatin. Somatic missense mutations in ERCC2 have been associated with improved survival and decreased recurrence in MIBC (muscle-invasive bladder cancer) patients^46,83^.

Direct repair via MGMT has been associated with lower rates of tumorigenesis in MGMT overexpressing mice^84^. Exploitation of direct repair has clear clinical implications in glioblastomas; in fact, a randomized clinical trial by Hegi et al.^85^ demonstrated that glioblastoma patients with a methylated MGMT promoter had better outcomes after treatment with temozolomide, an alkylating agent, although a smaller effect was still observed in patients without MGMT promoter methylation. The role of MGMT promoter methylation in therapeutic response has been investigated with conflicting findings, but overall the data suggest possible MGMT involvement in a variety of cancers including malignant melanoma, colorectal cancer, and lung cancer.

Alterations in *ATM* demonstrate synthetic lethality with *ATR* in preclinical models^86^ and more recently, with *BRCA1*^87^. *ATM* alterations have also been associated clinically with exceptional response to radiotherapy^88^ and additional analysis of exceptional responders may lead to novel synthetic lethal relationships that can be exploited for personalization of DNA damaging therapy.

### Synthetic viability as a potential mechanism of PARP inhibitor resistance

Synthetic viability is a mechanism of resistance to DNA damaging agents in which a reversion mutation mitigates existing DNA repair defects. A reversion mutation refers to the restoration of gene function, either partial or full, as a result of secondary mutations. Several studies have demonstrated that reversion mutations in BRCA1/2 result in drug insensitivity to platinum agents and PARP inhibitors in tumors that are initially PARP inhibitor sensitive. 53BP1 deficiency has also been shown to partially reverse the BRCA1-deficient phenotype. 53BP1 is a nuclear protein involved in DNA repair response, checkpoint control, and VDJ recombination; 53BP1-deficient cells demonstrate sensitivity to DNA damaging agents and ionizing radiation^89^. Furthermore, loss of 53BP1 or REV7 reverses PARP inhibitor sensitivity in BRCA-deficient cells, indicating that 53BP1 is synthetically viable with BRCA1 in preclinical studies^90,91^. Therefore, it is important that assessments of HR account for the possibility of reversion mutations, which favors the use of functional assays of DNA repair.

## Targeting the immune system in tumors with DNA repair defects

Multiple DNA repair pathways play a critical role in the development of the immune system^92^ and several play a critical role in oncogenesis. However, the mechanism of interaction between the DDR and the immune system during oncogenesis is only now starting to be realized and investigated. Defects in repair pathways lead to the development of specific lesions in the DNA sequence, and each of these can have their own unique effects on the tumor micro-environment, and more specifically the immune system’s response to a developing tumor. Alterations in DNA repair could influence either how the adaptive, innate, or both parts of the immune system respond to the underlying malignancy. Defects in DNA repair may influence the adaptive immune system by leading to an increased number of mutations, and subsequently increased number of neoantigens, which in turn increases the foreignness of a tumor (i.e., antigenicity), resulting in a higher probability of recognition by the tumor by the immune system. DNA repair could also influence how the innate immune system initially responds to a tumor and recruits the adaptive immune system to the site of malignancy. These are not mutually exclusive hypotheses and in fact both mechanisms likely play an important role in the interaction between tumor DNA repair defects and immune recognition and provide opportunities for therapeutic exploitation.

### Defects in DDR improve recognition of tumors by the adaptive immune system

**Fig. 3:**
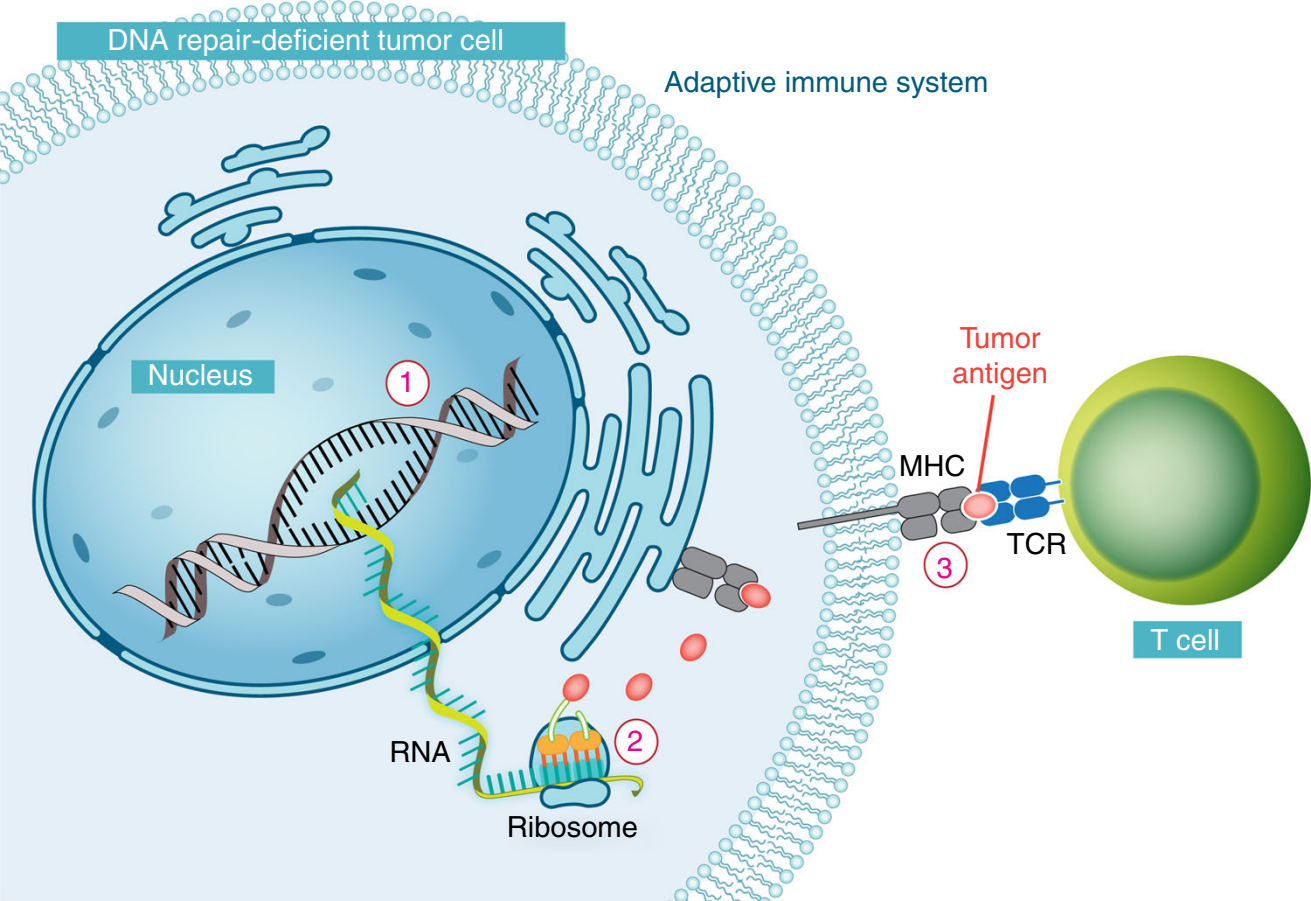
The neoantigen hypothesis: the adaptive immune system and DNA repair deficiencies in a DNA repair-deficient tumor cell. A non-synonymous mutation results in an amino acid change which yields a novel peptide which has the potential to be recognized as foreign by the immune system^94^. Defects in multiple DNA repair pathways can increase the number of non-synonymous mutations, including MMR, POLE/D, and even HR

For the adaptive immune system to eradicate a tumor, it must have some mechanism to recognize it as foreign and non-self^93^. Possibilities for antigens recognized by the immune system include cancer testis-antigens (i.e., genes normally only expressed in germline tissue, but aberrantly expressed in some cancer), tissue differentiation genes, overexpressed oncogenes, or neoantigens. Neoantigens are novel proteins that are most commonly a result of somatic mutations that are restricted to a tumor. That is, a non-synonymous mutation will result in a change in amino acid, which will subsequently result in a novel peptide that the immune system has not seen before, which has the potential to be recognized as foreign^94^ (Fig. 3). The advent of high-throughput sequencing technologies has now allowed for surveying the landscape of neoantigens in any particular tumor, and emerging preclinical evidence from murine models and human tumors suggests these play an important role in immune recognition^94^.

The number of neoantigens in a tumor is directly proportional to the number of non-synonymous mutations in a tumor, which in itself is known to vary by several orders of magnitude between and within different cancer types^5^. Defects in multiple DNA repair pathways can increase the number of non-synonymous mutations, including MMR, POLE/D, and even HR. Interestingly, mismatch repair-deficient tumors have long been known to induce a strong inflammatory response, which may in part be due to not only a significantly elevated number of non-synonymous mutations but also a large number of small insertion/deletions which result in frameshift mutations, which in turn produce highly novel (and very foreign) proteins^95^. HR-deficient tumors, although not as mutagenic as MMR deficiency, also result in an elevated number of non-synonymous mutations and have also been associated with increased immune infiltrate^96^. In fact, pan-cancer analysis has suggested that increasing mutation load is linearly related to increasing immune activity in the micro-environment of a tumor, suggesting that any elevation in mutation rate is likely to influence immune recognition^97^.

The advent of immune checkpoint blockade and the demonstration that the immune system can be harnessed effectively against cancer in a therapeutic setting has allowed this interaction between DNA repair and the immune system to be further explored. Two initial studies in melanoma with anti-CTLA-4 therapy and NSCLC with anti-PD-1 therapy both demonstrated improved overall survival in patients with higher mutation loads, supporting the idea that the immune system is more likely to recognize tumors with elevated neoantigens^98,99^. Subsequently, multiple groups have confirmed higher mutation loads are associated with an increased rate of response to checkpoint blockade in a variety of malignancies. A phase 2 study primarily in colorectal tumors, which have modest mutation load, demonstrated a pronounced improvement in overall survival and progression-free survival in patients who were MMR deficient, and in general had significantly more mutations than MMR-proficient tumors^100^. New data from Le et al.^101^ demonstrate similar findings across 12 cancer types, suggesting a broader application for PD-1 blockade. Further, in a preclinical murine model, isogenic introduction of an *MSH2* mutation resulted in marked improvements in immune response^102,103^. For MMR-deficient tumors, whether the amount of non-synonymous mutations or indels play an important role in response to therapy remains unclear; however, for some tumors, indels, which generate highly novel peptides, may play a more important role in immune recognition^104^. Pathways involving defective DSB repair and MMR are more likely to produce indels, whereas polymerase deficiencies and NER would more likely elevate non-synonymous mutations.

DDR-related therapeutics could be rationally combined with immune-therapeutics to try and exploit the neoantigen-based hypothesis or try to increase the antigencity of a tumor. One possibility includes using mutagenizing chemotherapeutics to introduce more neoantigens to facilitate immune recognition. Clinical trials in multiple disease sites with chemotherapeutics and/or PARP inhibitors plus immune checkpoint blockade using this rationale are ongoing. However, one issue with this approach is that mutagenizing agents are very likely to introduce subclonal mutations, which have been previously shown to be less effective in eliciting an immune response^105^. Alternatively, cytotoxic chemotherapeutics and IR could be used to facilitate antigen presentation by inducing tumor lysis, increasing major histocompatibility complex-I presentation, and/or altering the set of peptides presented^106^. Further, chemotherapy may facilitate epitope spreading or improve the response to sub-dominant antigens that were previously only weakly recognized^107^. Identifying differences between DNA repair-related therapeutics and their antigenicity-based consequences should facilitate a more rational approach to combining DNA-directed therapies with immunotherapies.

### Defects in DNA repair activate the innate immune system

**Fig. 4:**
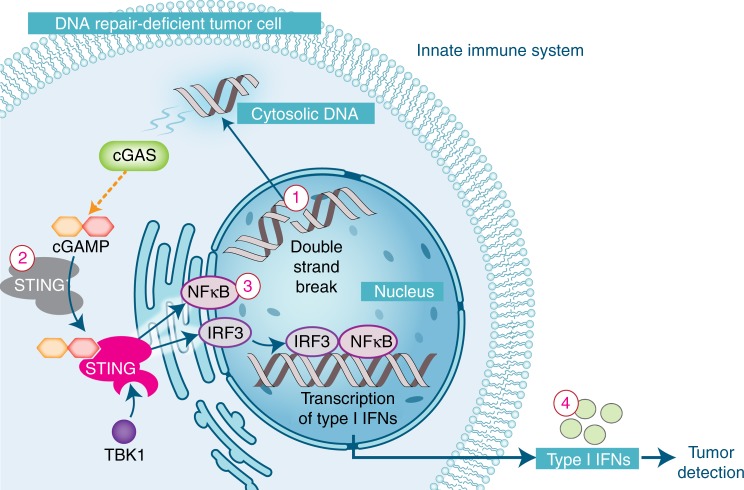
The STING hypothesis: activation of the innate immune system by DNA repair deficiencies in a DNA repair-deficient tumor cell. Cytosolic double-stranded DNA can be detected by cGAS which subsequently produces cGAMP (a cyclic dinucleotide), which in turn activates STING^119^. Upon activation, STING undergoes a conformational change, complexes with TANK-binding kinase 1 (TBK1) and relocates to peri-nuclear region. There, TBK1 phosphorylates IRF3 and NFKB, which translocate to the nucleus and lead to the expression of immune-related genes, including type I interferons^120^

Before the adaptive immune system can recognize a tumor as foreign, immune cells must be recruited to the site of a tumor, which typically occurs through the innate immune system. How the innate immune system senses the presence of a malignancy has remained perplexing, but recent data suggest that this recognition is often mediated by activation of Stimulator of Interferon Genes (STING) (Fig. 4)^108^. STING, a signaling molecule associated with the endoplasmic reticulum, was initially identified as playing a central role in generating an immune response to DNA-based viruses and bacteria by recognizing certain specific cytosolic DNA species, such as cyclic di-nucleotides^108,109^.

Recent evidence suggests that the STING pathway also plays an important role in leading to an innate immune response to malignancy. Typically, the innate immune system is active by release of danger-associated molecules, which could also include activation of Toll-like receptor pathways, cystolic RNA sensing, or extracellular adenosine triphosphate sensing, in addition to the STING pathway. Both syngeneic and carcinogen-induced models require dendritic cells with intact STING signaling, but not other innate immune signaling mechanisms, for efficient T-cell priming and subsequent immune recognition^110^. These data suggest that dying tumor cells are taken up by phagocytes and that DNA from these dying cells triggers a STING response, leading to an innate immune response.

In murine models, exogenously induced DNA damage with agents such as IR or certain chemotherapeutics induce a strong STING response^111,112^. Further, in a syngeneic MC38 murine tumor model, knockout of *TMEM173* (STING) significantly reduces tumor control after single-dose IR. Similar to exogenous DNA damage, inherent DNA repair defects in tumors may also increase production of cystolic DNA and appear to similarly trigger a STING response^113^. For example, fibroblasts from ataxia-telangiectasia (AT) patients demonstrated significantly higher levels of interferon-gamma genes and in AT-null mice, this effect appeared to be mediated by STING and result from accumulation of cytoplasmic DNA^113^. Interestingly, two recent reports have suggested that genome instability leads to cGAS (cyclic GMP-AMP synthase) localization to micronuclei with subsequent STING activation, which is dependent on cell cycle progression^114,115^. Several DSB repair deficiency defects are anticipated to result in elevated production of micronuclei and should be influenced by this effect.

In ovarian cancers, BRCA1/2-mutated tumors are associated with high tumor-infiltrating lymphocytes demonstrating improved prognosis, and in addition to elevated mutations would be anticipated to have more frequent micronuclei, leading to activation of the STING response^116^. In a murine genetically engineered model of *BRCA1* breast cancer, the combination of anti-CTLA-4 and anti-PD-1 therapy with cisplatin-based chemotherapy resulted in improved survival^117^. DNA damaging agents that produce double-strand breaks leading to cytosolic double-stranded DNA or the production of micronuclei could possibly synergize well with anti-PD-1 therapy by facilitating innate immune recognition. Studies with a broad spectrum of chemotherapeutics and more targeted DNA damaging agents are underway and should help elucidate whether this mechanism plays an important role in human tumors.

## Future directions

Further study of DNA repair-specific mutational signatures will have significant scientific and therapeutic implications. Identifying mutational signatures for individual DNA repair pathways will permit investigation into their prevalence in human cancers. Clear understanding of the contributory tumorigenic pathways will allow for expansion of precision therapies, through the mechanism of synthetic lethality or other novel therapeutic targets. Looking beyond DNA repair towards its interaction with other key cellular processes, particularly with the immune system, holds extraordinary potential for combination therapy.

### Data availability

All five authors contributed to the design and writing of the review paper.
